# COVID-19 und Public Health: Wissen, Einstellungen, Belastungen und Kommunikation in der Krise: Reflexionen zur Ausgangslage in der Bevölkerung und zu behördlichen Kommunikationsstrategien in der COVID-19-Pandemie

**DOI:** 10.1007/s00103-021-03293-1

**Published:** 2021-03-01

**Authors:** Anke Spura, Freia De Bock

**Affiliations:** 1 Leitung Referat 2-24 Fortbildung/Qualifizierung/Hochschulkooperation, Bundeszentrale für gesundheitliche Aufklärung (BZgA), Maarweg 149–161, 50825 Köln, Deutschland; 2 Leitung Abteilung 2 Effektivität und Effizienz der gesundheitlichen Aufklärung, Bundeszentrale für gesundheitliche Aufklärung (BZgA), Maarweg 149–161, 50825 Köln, Deutschland

Das vorliegende Themenheft „Wissen, Einstellungen, Belastungen und Kommunikation in der Krise“ ist das erste Heft einer vierteiligen Reihe „COVID-19 und Public Health“, die im Jahr 2021 erscheinen wird. Mit der Heftreihe soll ein erster systematischer Zwischenstand zu den Herausforderungen für Public Health in der Coronapandemie dargestellt werden, nachdem einzelne Diskussionspunkte (u. a. zu medialen Aspekten) bereits in vergangenen Heften aufgegriffen wurden.

Wir können nun bereits auf ein ganzes Jahr zurückblicken, das durch einen kollektiven, tiefgehenden und auch mit Belastungen einhergehenden Erkenntnis- und Lernprozess geprägt ist. Dieser Lernprozess betrifft die Herausgeberinstitutionen des Bundesgesundheitsblatts unmittelbar, da sie in das operative Krisenmanagement des Bundes einbezogen sind.

Ausgangspunkt für die Pandemie waren erste Beobachtungen in China zum Jahresende 2019. Im März 2020 wurde das Geschehen von der WHO als Pandemie definiert, es folgte die Feststellung einer epidemischen Lage von nationaler Tragweite durch den deutschen Bundestag. Anfänglich eine Gesundheitskrise mit epidemischem Charakter, entwickelten sich die Geschehnisse schnell zu einer gesamtgesellschaftlichen Herausforderung, welche beispielsweise das soziale und wirtschaftliche Leben tiefgreifend beeinflusst.

In dieser Situation größter Herausforderung war es immer Ziel der staatlichen Maßnahmen, den unerwünschten Folgen der COVID-19-Pandemie entgegenzuwirken. Güterabwägungen waren ständiger Begleiter der Pandemie in den Medien, der Politik, in Diskursen von Wissenschaft und Bundesverwaltung sowie bei Gesprächen allerorten in der Bevölkerung. Die staatliche Krisenkommunikation hatte daher nicht nur eine Rolle in der Ziel- und Maßnahmendefinition, sondern auch in der Diskursbegleitung und bei der Förderung von Akzeptanz und gesellschaftlichem Lernen, auch wenn nicht alles nach „Lehrbuch“ verlaufen ist.

Was lässt sich nun lernen? Welche wissenschaftlichen Daten haben wir ein Jahr nach Beginn der Pandemie? Welche Konzepte und Praktiken haben sich entwickelt? Die Herausgeberinstitutionen des Bundesgesundheitsblatts hoffen, dass der Zwischenbericht in dieser Reihe Sie mitnimmt in die Diskussion um den Entstehungsprozess politischer Maßnahmen und die gesellschaftliche Entwicklung in Deutschland unter dem Brennglas Corona. Dabei wird trotz aller Erkenntnisse, die wir im letzten Jahr gewonnen haben und zu denen die Themenhefte beitragen sollen, deutlich, dass Politik, Behörden und Wissenschaft weiterhin mit enormen Unsicherheiten umgehen und trotzdem Entscheidungen fällen müssen.

Das erste Themenheft dieser Reihe, das von der Bundeszentrale für gesundheitliche Aufklärung (BZgA) koordiniert wurde, möchte Reflexionen zur Ausgangslage in Bevölkerung und Gesellschaft im Jahr 2020 und zur behördlichen Krisenkommunikation anregen:

Trotz erheblicher Erkenntnissprünge in der klinischen Versorgung kann bis heute nicht auf ein einheitliches Therapieregime zurückgegriffen werden. Für die Bekämpfung der COVID-19-Erkrankung, die durch das SARS-CoV-2-Virus verursacht wird, bleiben daher vor allem die Mittel der Prävention. Anfang 2021 setzt die Gesellschaft ihre Hoffnung auf primäre Prävention durch Impfung. Die Entwicklung und Zulassung der Impfwirkstoffe und der Start der Impfkampagne zum Jahreswechsel stellen daher eine wichtige Zäsur dar.

Angesichts der zweiten Welle und der neuen Mutationen von SARS-CoV‑2 bleiben die bevölkerungsweiten nichtpharmazeutischen Interventionen (NPI) bis zur Erreichung eines ausreichenden Gemeinschaftsschutzes notwendig, um das Infektionsgeschehen einzudämmen. NPI können jedoch nur ihre Wirkung entfalten, wenn sie auf Verständnis, Akzeptanz und Beteiligung in der Bevölkerung treffen. Für alle Präventionsmaßnahmen ist daher Kommunikation der Dreh- und Angelpunkt.

Ausgangspunkt für bevölkerungsbezogene NPI ist, dass Erkenntnisse über Einstellungen in der Bevölkerung so vorliegen, dass Maßnahmen zielgruppen- und bedarfsorientiert ausgerichtet werden können. Die von *Eitze et al.* beschriebene COSMO-Studie gehörte zu den ersten Studien, die als wöchentlich wiederholte querschnittliche Onlinebefragung von jeweils 1000 Befragten ein Meinungsbild zur Verfügung stellen konnten. Der Wirkzusammenhang ist naheliegend: Die Zustimmung zu NPI und die Beteiligung daran sind gekoppelt an Vertrauenswerte. Vertrauen wird gestärkt, wenn die Sichtweisen und Lebenslagen aus der Bevölkerung in die Maßnahmenumsetzung einbezogen werden. Für das erste Halbjahr bescheinigt COSMO staatlichen Institutionen hohe Vertrauenswerte, aber Schwankungen der Vertrauenswerte zeigen, dass Vertrauen mühsam generiert und auch aufrecht gehalten werden muss.

Der Beitrag von *Bitzer et al.* untersucht die Inhalte von Internetpräsenzen unterschiedlichster Anbieter von Gesundheitsinformationen und -leistungen und klärt, inwiefern Menschen mit gesundheitlichen Beschwerden jenseits von COVID-19 in den ersten Monaten der Pandemie in Bezug auf die Inanspruchnahme von Versorgungsleistungen unterstützt wurden. Im Ergebnis informieren vorhandene Informations- und Beratungsangebote nur selten zu diesem Thema. Zur Sicherstellung der Versorgung und zur Erhöhung der Gesundheitskompetenz sollten solche Angebote entwickelt bzw. bekannt gemacht werden, u. U. auch über das nationale Gesundheitsportal www.gesund.bund.de.

Im Infektionsschutzgesetz, in der entsprechenden Verwaltungsvorschrift und im Nationalen Pandemieplan sind die staatlichen Kommunikationsaufgaben grundsätzlich definiert. Behörden wie die Bundeszentrale für gesundheitliche Aufklärung (BZgA), das Robert Koch-Institut (RKI) und das Paul-Ehrlich-Institut (PEI) sowie das Bundesinstitut für Arzneimittel und Medizinprodukte (BfArM) arbeiten in diesem Feld eng aufeinander abgestimmt. Die BZgA übernimmt beispielsweise die Aufgaben der gesundheitlichen Aufklärung für die Allgemeinbevölkerung, das RKI vor allem die der Kommunikation mit der Fachöffentlichkeit. Ihre Aufgabenerledigungen basieren sowohl auf kommunikationstheoretischen Grundannahmen als auch auf der bestmöglichen Evidenzlage. Wie sich das Vorgehen im Jahr 2020 darstellte, stellen für die BZgA *von Rüden et al.* und für die Risikokommunikation des RKI *Loss et al.* vor.

Der Diskussionsbeitrag von *Pfenninger et al.* schließt den ersten Themenbereich ab und reflektiert die Gefährdung von medizinischem Personal durch eine SARS-CoV-2-Infektion noch ohne einen verfügbaren Impfschutz.

Über das Jahr 2020 hinweg wurde sehr deutlich, dass die Pandemie nicht nur ein medizinisch-naturwissenschaftliches Problem darstellt, sondern auch Ausgangs- und Endpunkt für soziale Belastungen ist. In diesem Sinne vertieft der zweite Teil des Schwerpunktheftes, welche Folgen sich im Rückblick ergaben:

*Wahrendorf et al.* zeigen anhand ihrer Routinedatenauswertung von über 1,2 Mio. Versicherten einer gesetzlichen Krankenversicherung eindrücklich, wie sich Gesundheitsrisiken konkret entlang des sozialen Gradienten Erwerbstätigkeit manifestieren. So herausragend das Erleben dieser Pandemie scheint, so klar reproduziert sie bereits Bekanntes hinsichtlich gesundheitlicher Ungleichheit: Je geringer der Erwerbsstatus, umso höher die COVID-19-Hospitalisierungsrate – so lassen sich die Auswertungen zusammenfassen. Demnach tritt zu den Risikofaktoren für schwere Verläufe wie Alter oder Vorerkrankungen auch der sozioökonomische Status hinzu.

Weitere Folgen lassen sich hinsichtlich der psychischen Belastung in der Bevölkerung ausmachen. *Skoda et al.* untersuchten diese anhand einer Querschnittsstudie, die insgesamt 22.961 Menschen berücksichtigte. Die Studie weist insgesamt eine erhöhte psychische Belastungslage nach. Die psychische Belastung ist nicht mit der Angst vor einer SARS-CoV-2-Infektion gleichzusetzen, sondern insbesondere auf NPI oder wirtschaftliche Folgeentwicklungen bezogen und äußert sich mitunter erst nach einer akuten Schockphase zeitversetzt. Psychische Gesundheit wird in Postpandemiezeiten daher mit Sicherheit noch mehr als bisher ein wichtiges Feld für Prävention und Gesundheitsförderung sein.

Der Beitrag von *Welzel et al.* greift spezifisch die Folgen der Isolationsmaßnahmen für ältere Menschen auf, weil sie eine besonders hohe altersassoziierte Vulnerabilität für schwere COVID-19-Krankheitsverläufe mit sich bringen. Aus dieser Betrachtung wird deutlich, dass Krisenkommunikation möglichst an die Bedarfe und Ausgangslagen von zu differenzierenden Zielgruppen ausgerichtet werden muss.

Eine weitere besonders vulnerable Zielgruppe sind Geflüchtete. Wie sich die Coronapandemie auf die Situation in den Gemeinschaftsunterkünften und Erstaufnahmeeinrichtungen auswirkte, zeigen *Biddle et al.* in einer qualitativen Befragung von insgesamt 48 Akteuren in den Aufnahmebehörden. Aus den Ergebnissen wird deutlich, dass Pandemieeindämmung und Krisenkommunikation nicht nur Aufgaben von Bundes- und Landesseite sind, sondern dass Akteure auf regionaler und kommunaler Ebene wichtige Teile der Wirkungskette von Kommunikation in der Pandemie sein können.

Wir wünschen Ihnen viel Freude und Lernen beim Lesen dieses ersten Heftes unserer Reihe.

Ihre

Anke Spura

und

Freia De Bock

